# Six Long-Standing Questions about Antibiotic Prophylaxis in Surgery

**DOI:** 10.3390/antibiotics12050908

**Published:** 2023-05-15

**Authors:** Massimo Sartelli, Marja A. Boermeester, Miguel Cainzos, Federico Coccolini, Stijn W. de Jonge, Kemal Rasa, E. Patchen Dellinger, Deborah A. McNamara, Donald E. Fry, Yunfeng Cui, Samir Delibegovic, Zaza Demetrashvili, Belinda De Simone, George Gkiokas, Timothy C. Hardcastle, Kamal M. F. Itani, Arda Isik, Francesco Maria Labricciosa, Varut Lohsiriwat, Sanjay Marwah, Tadeja Pintar, Jennifer Rickard, Vishal G. Shelat, Fausto Catena, Philip S. Barie

**Affiliations:** 1 Department of Surgery, Macerata Hospital, 62100 Macerata, Italy; 2 Department of Surgery, Amsterdam University Medical Center, University of Amsterdam, 1105AZ Amsterdam, The Netherlands; 3 Department of Surgery, Hospital Clínico Universitario, 15706 Santiago de Compostela, Spain; 4 Department of Emergency and Trauma Surgery, Pisa University Hospital, University of Pisa, 55126 Pisa, Italy; 5 Department of Surgery, Anadolu Medical Center, 41200 Kocaeli, Turkey; 6 Department of Surgery, University of Washington, Seattle, WA 98195, USA; 7 Department of Colorectal Surgery, Beaumont Hospital, D09V2N0 Dublin, Ireland; 8 Department of Surgery, Northwestern University, Chicago, IL 60208, USA; 9 Department of Surgery, Tianjin Nankai Hospital, Nankai Clinical School of Medicine, Tianjin Medical University, Tianjin 300100, China; 10 Department of Surgery, University Clinical Center of Tuzla, 75000 Tuzla, Bosnia and Herzegovina; 11 Department General Surgery, Kipshidze Central University Hospital, Tbilisi 0162, Georgia; 12 Department of Emergency and Metabolic Minimally Invasive Surgery, Centre Hospitalier Intercommunal de Poissy/Saint Germain en Laye, 78300 Poissy CEDEX, France; 13 Second Department of Surgery, Aretaieion University Hospital, National and Kapodistrian University of Athens, 10679 Athens, Greece; 14 Department of Surgery, Nelson R. Mandela School of Clinical Medicine, University of KwaZulu-Natal, Mayville 4058, South Africa; 15 Department of Surgery, VA Boston Health Care System, Boston University and Harvard Medical School, Boston, MA 02118, USA; 16 Division of General Surgery, School of Medicine, Istanbul Medeniyet University, 34700 Istanbul, Turkey; 17 Global Alliance for Infections in Surgery, 62100 Macerata, Italy; 18 Department of Surgery, Faculty of Medicine Siriraj Hospital, Mahidol University, Bangkok 10700, Thailand; 19 Department of Surgery, BDS Post-Graduate Institute of Medical Sciences, Rohtak 124001, India; 20 Department of Abdominal Surgery, University Medical Centre Ljubljana, 1000 Ljubljana, Slovenia; 21 Department of Surgery, University of Minnesota, Minneapolis, MN 55455, USA; 22 Department of Hepato-Pancreatic-Biliary Surgery, Tan Tok Seng Hospital, Singapore 308433, Singapore; 23 Department of Surgery, “Bufalini” Hospital, 47023 Cesena, Italy; 24 Department of Surgery, Weill Cornell Medicine, E. Northport, New York, NY 11731, USA

**Keywords:** surgical site infections, surgical antibiotic prophylaxis, healthcare-associated infections

## Abstract

Surgical site infections (SSIs) are the most common adverse event occurring in surgical patients. Optimal prevention of SSIs requires the bundled integration of a variety of measures before, during, and after surgery. Surgical antibiotic prophylaxis (SAP) is an effective measure for preventing SSIs. It aims to counteract the inevitable introduction of bacteria that colonize skin or mucosa into the surgical site during the intervention. This document aims to guide surgeons in appropriate administration of SAP by addressing six key questions. The expert panel identifies a list of principles in response to these questions that every surgeon around the world should always respect in administering SAP.

## 1. Introduction

Healthcare-associated infections (HAIs) are those infections that patients acquire while receiving healthcare and that are neither present nor incubating at the time of admission [1].

Surgical site infections (SSIs), central-line-associated blood stream infection, catheter-associated urinary tract infections, hospital-acquired pneumonia, ventilator-associated pneumonia, and *Clostridioides difficile* infections (CDI) represent the most common HAIs. They can lead to adverse patient outcomes and are responsible for high costs for patients and healthcare systems [2].

Many HAIs are preventable, and therefore, these infections can be considered an important indicator of the quality of care provided to patients [3]. SSIs are the most common HAIs across the surgical pathway, representing and the most common adverse event occurring in surgical patients [1].

The bacteria isolated from SSIs [4] may differ depending on the site and degree of colonization or contamination of the surgical procedure. In patients undergoing clean (Class I) surgery (e.g., breast, thyroid) in which the surgical site is limited to skin and subjacent soft tissue (the gastrointestinal, genitourinary, or respiratory tracts have not been opened,) bacteria from the patients’ skin flora, usually commensal gram-positive cocci, are isolated most frequently. However, the skin of some specific areas of the body, such as the groin and perineum, can also be colonized by intestinal or genitourinary tract flora, typically aerobic or facultative gram-negative bacilli. In clean/contaminated (Class II) operations—in which the gastrointestinal, genitourinary, or respiratory tracts have been opened under controlled circumstances (e.g., cholecystectomy, bowel resection/anastomosis)—and contaminated (Class III) surgical procedures (e.g., enterotomy during adhesiolysis)—in which the gastrointestinal, genitourinary or respiratory tracts have been opened under uncontrolled circumstances—endogenous microflora (aerobic and anaerobic bacteria and yeast or fungi) of the surgically opened hollow viscus are isolated most frequently.

Prevention of SSIs requires the bundled integration of a variety of measures before, during, and after surgery. The World Health Organization (WHO) [5,6,7] and the U. S. Centers for Disease Control and Prevention (CDC) [8] published their guidelines for the prevention of SSIs in 2016 and 2017, respectively. In 2016, the American College of Surgeons (ACS) and the Surgical Infection Society (SIS) also published collaborative guidelines on SSI prevention [9]. In 2019, the National Institute for Health and Care Excellence (NICE) of the United Kingdom updated its guidelines for the prevention and management of SSIs [10].

As part of the ensemble of measures to prevent SSI, appropriate surgical antibiotic prophylaxis (SAP) is an effective measure for preventing SSIs. It aims to counteract the inevitable introduction of bacteria that colonize skin or mucosa into the surgical site during the intervention. Inappropriate SAP has been documented for nearly 30 years [11], yet robust evidence for the efficacy of single-dose SAP compared to multiple doses has existed for nearly as long [12], and calls for rigor in the utilization of SAP have been published for more than 20 years [13,14]. The advent of antimicrobial stewardship has led to calls for surgeons to adopt its principles and embrace evidence-based prescription of antimicrobials [15], but evidence of efficacy is mixed [16,17], and reports continue to indicate that adoption is suboptimal due to cultural resistance to change, limited resources, failure to prioritize due to other competing demands, and lack of resolve from leadership [18,19]. It is long past time for surgeons to take ownership and leadership and administer SAP correctly.

## 2. Methods

In December 2022 the Global Alliance for Infections in Surgery instituted an international expert panel to develop a shared narrative review regarding the principles of SAP, involving twenty-four experts worldwide with great experience in this field.

An extensive search of the literature was conducted using the PubMed^®^/MEDLINE (National Library of Medicine, Bethesda, MD, USA) and Google Scholar (Alphabet, Inc., Mountain View, CA, USA) databases. The time limit for the review was between 1 January 1996 and 31 March 2023.

The following list of search terms was used: “antibiotic prophylaxis”, “perioperative antibiotic prophylaxis”, “surgical antibiotic prophylaxis”, “surgical wound infection”, “surgical site infection”, “SSI”, “SSIs”, “surgical infection”, “post-operative wound infection”. Articles in languages other than English were excluded.

A reviewer screened titles and abstracts of gathered references for potentially relevant studies. Full texts of all eligible papers were retrieved, and each reference list was reviewed for any omitted studies. Afterwards, the first draft was shared with the international expert panel. The panel reviewed the document and completed it. The resulting document was submitted again to all members of the panel, reviewed, and finally approved.

This narrative review written by surgeons aims to inform and guide surgeons worldwide in the correct administration of SAP by addressing six key questions. The expert panel also identified a list of principles in response to these questions that every surgeon worldwide should always respect in administering SAP.

## 3. Surgical Antibiotic Prophylaxis

Antibiotic use is under scrutiny due to concerns about the emergence of antimicrobial resistance (AMR) and other hazardous side effects [20,21]. Longer exposure to antibiotics has been associated with an increased risk of antimicrobial resistance, CDI, and acute kidney injury [22,23,24], while avoiding postoperative continuation of antibiotic prophylaxis has been associated with reduced risk of CDI [25].

The role of SAP in preventing SSIs has been well described in the literature [3]. Bowater et al. [26] analyzed data from 21 meta-analyses based on randomized controlled trials (RCTs) that included 48,909 patients from 250 hospitals, demonstrating that the administration of SAP has a significant effect on the prevention of SSIs irrespective of the contamination of the wound (wound class) and type of surgical procedure (relative risk reduction 18–78%.)

In 2008, Whitman et al. [27] established the importance of protocolized administration of SAP. The authors reviewed SAP administration retrospectively in 1622 consecutive patients to determine whether the choice of agent, timing of administration, and postoperative cessation of antibiotics were appropriate. Despite hospital-wide education, improving compliance with evidence-based recommendations for SAP required processes that “forced” surgeon behavior [22].

SAP can alter patients’ intestinal microbiota, leading to opportunistic infections such as CDI, and the overgrowth of antibiotic-resistant bacteria and fungi. Risk factors for the development of CDI include longer duration of SAP as well as environmental factors such as standards of hygiene, hand washing, and access to isolation facilities. The contribution of SAP to CDI is not clear, with rates of between 0.2% to 8.0% in patients who have received SAP depending on the procedure and patient-related factors. Limiting the duration of SAP to a single preoperative dose can reduce the risk of CDI [28] but cannot eliminate it entirely. Although the misuse and overuse of antibiotics are the main drivers of AMR [29] and are a result of the large numbers of surgical procedures, SAP is among the highest-volume categories of antibiotic use within health systems globally [30], and the effects of incorrect SAP on the spread of AMR have been studied insufficiently. A prolonged course of SAP resulted in a greater risk of acquiring resistant bacteria after cardiovascular surgery in an observational 4-year cohort study at a tertiary care center [24]. Likewise, prolongation of SAP in a cohort of critically ill surgical patients increased the risk of blood stream infection with multi-drug-resistant (MDR) bacteria [31].

Ideally, an appropriate SAP should respect several characteristics (Table 1) [32]. Moreover, the selected antibiotic for SAP should have some basic properties (Table 2) [32].

Joint guidelines for SAP in surgical procedures were revised and updated in 2013 by the American Society of Health-System Pharmacists (ASHP), the Infectious Diseases Society of America (IDSA), the Surgical Infection Society (SIS), and the Society for Healthcare Epidemiology of America (SHEA) [33].

Although clinical practice guidelines for SAP have existed for a decade, high rates of inappropriate prescribing practices are still common [18,19,34] and can contribute to suboptimal patient outcomes, cause adverse effects, and be a driver of AMR [35]. Continuation of SAP is still very common across the world. A recent global point prevalence study revealed that the percentage of patients that receive SAP for more than one day ranged from 29.5% in Western Europe to 92.5% in Africa [35].

**Figure 1:**
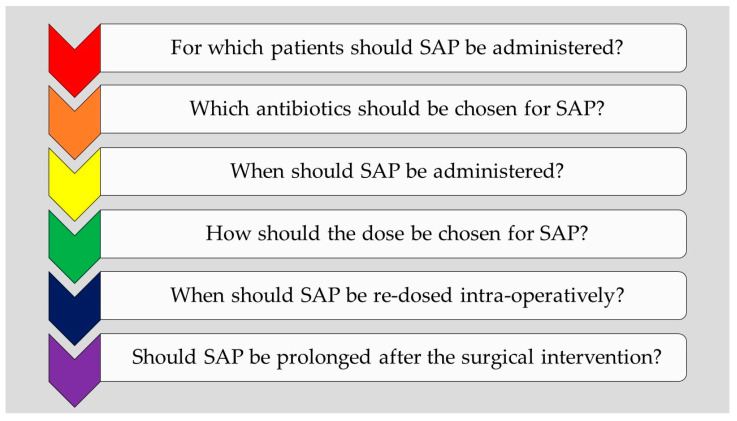
Six key questions regarding the correct approach to surgical antibiotic prophylaxis (SAP).

This document aims to guide surgeons in administering correctly SAP by answering six questions (Figure 1). In considering these questions, it is also essential to understand that SAP does not replace correct infection prevention and control measures before, during, and after operation.

### 3.1. For Which Patients Should SAP Be Administered?

SAP is suggested in surgical procedures associated with a high incidence of SSIs, such as most clean-contaminated and contaminated surgical procedures. It may also be indicated in certain clean procedures where SSIs, even if unlikely, may have devastating consequences as in procedures with prosthetic implants. Other potential candidates include patients with medical conditions associated with a higher risk of SSI—including immunocompromised patients (e.g., neutropenia, therapeutic immunosuppression)—or patients with American Society of Anesthesiologists (ASA) score > 3 or obesity [29].

Obesity has been related to an increased risk of SSI. In a 2016 analysis of the ACS National Surgical Quality Improvement Program (NSQIP) database for 2011 [36], it was demonstrated that obese patients have significantly greater SSI rates in clean and clean-contaminated interventions but not in contaminated interventions. For dirty surgery (Class IV), antibiotic therapy is indicated, not SAP. Logistic regression demonstrated that obesity is independently associated with SSI in clean (obesity: odds ratio (OR) 1.757; morbid obesity: OR 2.544; both, *p* < 0.001) and clean-contaminated cases (obesity: OR 1.239; morbid obesity: OR 1.287, *p* < 0.001). The role of SAP in patients undergoing open groin herniorrhaphy or hernioplasty remains uncertain owing to conflicting results of generally low evidence quality [37,38,39,40,41,42]. The 2018 International Guidelines recommended SAP in open-groin mesh repair in any patient in a high-risk environment [43].

### 3.2. Which Antibiotics Should Be Chosen for SAP?

The antibiotic of choice for SAP should be active against the common bacteria causing SSIs after the specific procedure. SSIs following clean procedures are usually due to gram-positive coccal commensal skin flora, including *Staphylococcus aureus* or coagulase-negative staphylococci. Clean-contaminated (Class II) and contaminated (Class III) incisions may harbor various other bacteria depending on the flora of the specific location (skin, mucosa) incised, such as *Escherichia coli* or other *Enterobacteriales* or *Clostridiales*.

It is important to choose antibiotics with the narrowest spectrum of activity required and thus to have knowledge of the microbiology of SSI at specific body sites. Broad-spectrum antibiotics should not be used for SAP (with a single exception—ertapenem) [44,45,46], as one may be required later if a patient develops an MDR postoperative infection. However, if SAP is appropriately parsimonious, it is unlikely for the SSI pathogen to be an MDR organism. Detailed recommendations regarding antibiotic choice are available in the ASHP/IDSA/SIS/SHEA joint guidelines [33].

The antibiotics used most commonly for SAP are first- and second-generation cephalosporins, such as cefazolin, cefuroxime, cefoxitin, or the combination of cefazolin plus metronidazole [47]. However, owing to decreasing activity against anaerobic and aerobic gram-negative bacilli and a short half-life, cefoxitin—an intravenous second-generation cephalosporin—is now discouraged for prophylaxis of elective colorectal surgery compared to cefazolin plus metronidazole [48,49,50,51]. Cefazolin, for most procedures, is the drug of choice for SAP [33]. It has been most widely studied and has proven efficacy, a favorable pharmacokinetic (PK) profile, an appropriate narrow spectrum of activity, reasonable safety, and low cost.

Routine use of vancomycin for SAP is not generally recommended. Vancomycin can be considered for SAP in patients with known methicillin-resistant *S. aureus* (MRSA) colonization or who are likely to have had recent exposure to MRSA, such as dialysis patients, patients with recent hospitalization, or patients residing in long-term care facilities. However, what constitutes “high” risk is debated. It may be logical to provide SAP with an agent active against MRSA for any patient colonized with MRSA for a soft tissue procedure who also undergoes a decolonization protocol (often topical nasal mupirocin plus chlorhexidine bathing) [52,53], but efficacy has been shown only for cardiac and orthopedic surgery (where bone is also usually incised) [54]. Moreover, even if vancomycin SAP is recommended when the risk for MRSA is perceived to be high, note that it is less effective than a first-generation cephalosporin for preventing SSIs caused by methicillin-susceptible *S. aureus* [55].

Allergy history to beta-lactam antibiotics is important to consider when selecting SAP, although most patients who sustain an anaphylactoid response (immunoglobulin (Ig)E-mediated, such as anaphylaxis, urticaria, bronchospasm, or angioedema; non-IgE-mediated, including Stevens–Johnson syndrome, toxic epidermal necrolysis, or other drug-induced cutaneous hypersensitivity reactions) to a beta-lactam report no prior history. Patients should be questioned carefully before the administration of SAP about their history of antibiotic hypersensitivity to determine whether a true allergy exists. Although by history, as many as 10% of patients will report allergy to penicillin, the incidence of serious adverse reactions is far lower (well under 1%) [56]. Moreover, the cross-reactivity of patients between penicillin and cephalosporin or carbapenem hypersensitivity is < 5%.

Patients with a history of anaphylactoid penicillin allergy should not receive any beta-lactam antibiotic for SAP [33], but most patients with unknown, dubious, or non-anaphylactoid reactions (e.g., maculopapular rash), to penicillin may receive a cefazolin safely [57] after appropriate informed consent. Cefazolin, in particular, possesses an R1 side chain that is distinct from other cephalosporins and beta-lactam antibiotics. Therefore, cross-reactivity with penicillin or other beta-lactams is not expected [58,59,60]. Clindamycin or vancomycin are alternative agents for SAP when cefazolin would otherwise be chosen but is contraindicated, but the alternatives provide zero gram-negative spectrum of activity, and SSI rates may be as much as 50% higher as a result [61].

Currently, there is limited guidance on selecting appropriate SAP in patients colonized with MDR bacteria due to a paucity of published data. The question of what antibiotic to use for patients known to be colonized or recently infected with MDR bacteria is complex to resolve and cannot be generalized to all patients [62]. Whether SAP should be prescribed to cover MDR bacteria depends on many factors, including their antibiotic susceptibility profile, the host, the procedure to be performed, and the proximity of the bacterial reservoir to the operative site. Pathogen-specific SAP in a patient with prior infection or colonization with a gram-negative MDR pathogen may not be necessary for a purely soft tissue operation but may be considered if the gastrointestinal tract or other mucosa-lined organ is to be opened.

Although a vast majority of patients who receive SAP do so intravenously, alternative routes of administration have been described for selective use, including oral, topical (by powder, ointment, or paste onto the open incision), or by addition to irrigation fluid. Oral preoperative administration is the only alternative route that may ensure adequate tissue concentrations at the time of incision. Desirable PK characteristics of any agent used for an oral SAP regimen include high oral bioavailability and a long half-life, which are met in contemporary practice by fluoroquinolones, but this comes at the sacrifice of an overarching desirable characteristic, i.e., narrow spectrum of activity. Single-dose oral SAP has been demonstrated most successfully in urology whether for endourologic procedure or transrectal prostate biopsy [63].

Topical antimicrobial agents remain popular despite lacking evidence of efficacy. Thirteen RCTs comparing topical antibiotics with non-antibiotic agents in patients with clean and clean-contaminated surgical incisions, published before 30 September 2020, were evaluated through meta-analysis [64]. There were no significant differences between topical antibiotics and non-antibiotic agents in terms of SSIs incidence and contact dermatitis (the primary safety signal).

Intraoperative wound irrigation is very common despite the lack of high-quality data supporting its use [65]. The practice is particularly popular in neurosurgery, plastic/reconstructive surgery, and orthopedics, using cefazolin, kanamycin, vancomycin, or—formerly—bacitracin, the latter of which was withdrawn by the U.S. Food and Drug Administration (FDA) owing to nephrotoxicity and anaphylaxis. In FDA parlance, use for intraoperative irrigation of surgical incisions to prevent infections SSIs is considered off-label. To date, the only FDA-approved products for wound irrigation include sterile normal saline, sterile water, and sterile water and 0.05% chlorhexidine gluconate in a specific medical device. Topical antiseptics offer an alternative, including dilute chlorhexidine gluconate (0.05%) or povidone-iodine (1%). A 2017 meta-analysis [66] of 21 studies found low-quality evidence that demonstrated a benefit of incisional irrigation with an aqueous povidone-iodine solution in clean and clean-contaminated incisions (OR 0.31; 95% CI 0.13–0.73; *p* = 0.007), but antibiotic irrigation had no effect in reducing SSIs (OR 1.16; 95% CI 0.64–2.12; *p* = 0.63). Considering the low-quality of the data and a lack of evidence of efficacy, safety, or cost-effectiveness, intraoperative antimicrobial irrigation cannot be recommended.

Oral antibiotic bowel preparation (oABP) for elective colon surgery merits special consideration. For most patients, oABP is prescribed in addition to mechanical bowel preparation (mBP) and intravenous (IV) SAP, leading to a long-standing debate as to whether reduced SSI rates were related to oABP or IV SAP in combination with elimination of bulk feces and reduced bacterial colonization [67]. Although the use of oABP in combination with mBP is a tactic employed widely in North America, it is less common across Europe, perhaps because Enhanced Recovery After Surgery (ERAS^®^) protocols exclude routine mBP [68,69]. Whereas mBP alone is now considered ineffective for reduction of SSI and anastomotic leak [70], several meta-analyses [71,72] now favor a combination of oABP with mBP the day before elective colorectal surgery in addition to IV SAP just prior to operation. Moreover, mBP improves laparoscopic surgical viewing and facilitates intraoperative manipulation of the bowel in minimally invasive surgery [73].

A frequentist random-effects network meta-analysis was conducted to estimate the network effects comparing multiple methods of bowel preparation (mBP, oABP alone, mBP + oABP, or no preparation) with regards to incidence of SSIs [74]. Included were 48 studies with 13,611 patients. Compared to no preparation, combined direct and indirect network estimates showed risk ratios (RRs) for SSI of 0.57 (95% CI 0.45–0.72) for mBP + oABP, 0.68 (95% CI 0.49–0.95) for oABP alone, and 1.05 (95% CI 0.87–1.26) for mBP. The RR for mBP + oABP compared to oABP alone was 0.84 (95% CI 0.60–1.19); in sensitivity analysis of mainly laparoscopic procedures, the effect of mBP + oABP was significant (RR 0.56; 95% CI 0.31–0.99). In this analysis, both mBP + oABP and oABP alone were comparably effective in the prevention of SSI, but the evidence is uncertain about the relative benefit of mBP + oABP compared to oABP alone.

An updated Cochrane meta-analysis [75] included 21 RCTs (5264 adult patients) undergoing elective colorectal surgery, comparing combined mBP plus oABP (mBP + oABP) with either mBP alone, oABP alone, or no bowel preparation whatsoever (nBP). Studies lacking perioperative IV SAP were excluded. Seventeen trials compared mBP + oABP with mBP alone. Compared with mBP alone, mBP + oABP reduced the incidence of SSI by 44% (RR 0.56, 95% CI 0.42, 0.74), and the risk of anastomotic leakage (causing organ-space SSI) was reduced by 40% (RR 0.60, 95% CI 0.36, 0.99). No difference was found in mortality risk (RR 0.87, 95% CI 0.27, 2.82), postoperative ileus (RR 0.89, 95% CI 0.59, 1.32), or length of hospital stay (MD − 0.19, 95% CI − 1.81, 1.44.) Three trials compared mBP + oABP to oABP alone. No difference was demonstrated in terms of SSI (RR 0.87, 95% CI 0.34, 2.21), anastomotic leakage (RR 0.84, 95% CI 0.21, 3.45), mortality (RR 1.02, 95% CI 0.30, 3.50), or incidence of postoperative ileus (RR 1.25, 95% CI 0.68, 2.33.) Based on moderate-certainty evidence, the authors concluded that mBP + oABP is more effective than mBP alone in preventing postoperative SSIs, but whether oABP alone is equivalent to mBP + oABP needs further clarification in light of low- to very-low-certainty evidence.

A major limitation of this body of evidence is substantial heterogeneity concerning choice of antibiotic and duration of SAP. Agents, dosages, and timing vary among studies, making the results difficult to generalize. No clear evidence exists as to which is the preferred oral agent and dosage for oABP before colorectal surgery.

### 3.3. When Should SAP Be Administered?

An adequate concentration of the antibiotic must be present at the surgical site throughout the intervention, at (just before) the time of incision and for the duration of the procedure, that exceeds the minimum inhibitory concentration (MIC) for the likely bacteria associated with the intervention. WHO global guidelines [7] recommend SAP administration prior to the surgical incision when indicated. These guidelines also recommend SAP administration within 120 min before the surgical incision, based on the half-life of the antibiotics.

In a 2017 meta-analysis that evaluated the correct timing of SAP and compared several different time intervals of administration [76], and 14 studies (54,552 patients), all observational, were included. There were no significant differences when SAP was administered 120–60 min before surgical incision compared with administration 60–0 min beforehand. Studies investigating time intervals shorter than 60 min reported contradictory results. The risk of SSIs was doubled when SAP was given after the incision was made (odds ratio (OR) 1.89; 95% CI 1.05–3.40)), and five-fold higher when SAP was given more than 120 min before the incision (OR 5.26; 95% CI 3.29–8.39). Also in 2017, Weber et al. [77] evaluated using RCT the optimal timing of SAP, which consisted of a single IV infusion of cefuroxime 1.5 g (a short-half-life second-generation cephalosporin) plus metronidazole 500 mg IV in colorectal surgery. Among 5580 patients, 2798 patients were assigned randomly to SAP 30–75 min before incision, whereas 2782 patients were assigned to SAP 0–30 min before incision. 5175 patients were retained for analysis. The authors found no significant differences between the groups (OR 0.93, 95% CI 0.72–1.21, *p* = 0.601).

Although the data indicate that SAP should be administered within 120 min before incision, according to antibiotic PKs the optimal time for preoperative SAP is within 60 min prior to incision for most antibiotics, including cefazolin and other first/second-generation cephalosporins. By doing so, tissue antibiotic concentrations are sufficient for operations at least four hours in duration, rendering moot the decision as to whether intraoperative redosing is required. Cefazolin in particular is safe to administer as a bolus dose immediately before incision. Some parenteral agents, including fluoroquinolones and vancomycin, require administration over at least one hour.

### 3.4. How Should the Dose Be Chosen for SAP?

Dosing considerations are moot for most patients, who have normal organ function (especially renal function) and who are not undergoing long-duration operations. The dose chosen should be the same as is used therapeutically [33]. Dosing considerations must be addressed for special populations, which include obese patients and possibly patients undergoing kidney or (less likely) liver transplantation.

With respect to obesity, PKs (especially tissue penetration/volume of distribution) vary for individual antibiotics depending on whether the agent is hydrophilic or lipophilic [78,79]. As an example [79], cefazolin, given as a single 2 g IV bolus 3–5 min before skin incision and provides protective cefazolin concentrations (>MIC of methicillin-susceptible *S. aureus*) for 4.8 h, longer than the duration of most bariatric procedures (regardless of body mass index, thus obviating the need for intraoperative redosing at 4 h. Because cefazolin is water-soluble (volume of distribution (Vd) = 0.2 L/Kg) [80], penetration into adipose tissue is not dose-dependent. Therefore, extremely high-dose cefazolin (i.e., 3–4 g) is unnecessary [78,79,81,82]. In contrast, cefoxitin is not as hydrophilic (Vd = 12 L/Kg) [83,84], and higher doses may be required. In bariatric surgery, doses as high as 4–6 g given as SAP often achieve PK dosing targets [85,86,87].

Ertapenem has an even higher Vd (~60 L/Kg) [88] owing to its long half-life and consequent once-daily dosing as either prophylaxis or therapy. Data indicate that a 1 g dose of ertapenem is effective SAP for trauma laparotomy [89] as well as elective colorectal surgery. Data are mixed with regard to dosing in obesity. Some data indicate that a single dose of ertapenem 1 g may be sufficient for SAP in obesity surgery of up to four hours, regardless of body mass index [90]. Other data indicate that a 1 g dose may be insufficient for obese females [91].

Despite reports of efficacy [92,93], there is no role for cefepime (a fourth-generation cephalosporin) or any third-generation cephalosporin as SAP. However, PK data do exist. In obese patients with normal renal function, Rich et al. found that the free concentration above the MIC (fT > MIC) for 60% of the dosing interval was determined to be 10.12 h, including time for infusion [94]. Based on their analysis, a cefepime dose of 2 g for SAP should be repeated every 8 h during prolonged surgery to maintain an adequate fT > MIC.

Unfortunately, data are sparse regarding the administration of SAP in solid organ transplantation (SOT) [95,96]. SOT recipients are at high risk for early postoperative infectious complications due to the complexity of surgical procedures, end-stage organ dysfunction, therapeutic immunosuppression in the post-transplant period, and the frequent need for reoperation in the postoperative period [97]. SOT patients are also at high risk for infections caused by MDR pathogens. Moreover, SOT recipients may be exposed to unique circumstances, such as infections from contaminated preservation fluid [98] and donor-derived infections. Recognition of these risks may lead to liberalized use of SAP in the SOT setting, but perceived overuse of SAP in SOT has led to calls for antibiotic stewardship [99]. The need for stewardship is underscored by a drug utilization review from a large transplantation center in the Middle East [100]. The study included 54 liver recipients and 163 kidney recipients. Compliance rates for the choice of antibiotic were acceptable (~80%) for both liver and kidney transplantation. High compliance was observed for duration of antibiotic exposure after kidney transplantation but not liver transplantation. Compliance in terms of dosing and administration time was poor in both groups. Only a single RCT of SAP for renal transplantation was published in the literature; single-dose prophylaxis was as effective for prevention of SSI as a multi-dose regimen [101]. Because most antimicrobial agents are excreted by the kidney, there will be no clearance of drug until the graft is perfused; unless the graft is from a live donor, and thus more likely to function immediately, delayed graft function may result in no need for intraoperative redosing at all. Cefazolin is recommended by the ASHP/IDSA/SIS/SHEA joint guidelines [33] for SAP of kidney or kidney–pancreas transplantation.

There are even fewer data regarding SAP of orthotopic liver transplantation (OLT) however, drug clearance is not usually an issue because most agents used for SAP are not affected by hepatic impairment unless liver failure has resulted in hepato-renal syndrome with acute kidney injury. A single “pilot” trial of SAP has been published [102], including 102 OLT recipients randomized equally to receive either intraoperative SAP only or 72 h of perioperative SAP. The rate of SSI was 19%, compared with 27% (*p* = 0.36). The results from this study suggest that it is acceptable for OLT recipients to receive intraoperative antibiotics alone, but additional RCTs of adequate power are needed for SAP of OLT. The ASHP/IDSA/SIS/SHEA joint guidelines [33] recommend piperacillin-tazobactam or cefotaxime + ampicillin prophylaxis or, alternatively, clindamycin or vancomycin + aminoglycoside or aztreonam or fluoroquinolone for patients with a β-lactam allergy.

### 3.5. When Should SAP Be Redosed Intraoperatively?

Although in 2017 the CDC guideline [8] did not identify sufficient high-quality evidence to evaluate the risk, the benefit of intraoperative redosing of SAP for the prevention of SSIs. From the standpoint of PKs, an additional intraoperative dose should be administered for procedures exceeding two half-lives of the antibiotic chosen [33] and again a third time if that time interval is reached again later during a long operation. For operations with substantial blood loss (>1.5 L), the data are even less clear.

Many of the antibiotics used prescribed as SAP have relatively short half-lives (1–2 h) [33]. It may therefore be logical to repeat an additional antibiotic dose when surgery lasts more than 2–4 h, based on the antibiotic, to ensure adequate serum and tissue concentrations. In the case of cefazolin, which has a half-life of about 2 h, an additional intraoperative dose should be repeated after about 4 h [33,103]. Otherwise, in the case of cefoxitin, which has a very short half-life of 40–60 min [28], a subsequent intraoperative dose should be repeated after about 2 h.

The importance of intraoperative SAP redosing was confirmed by a recent meta-analysis [103] that included two RCTs and eight cohort studies, totaling 9470 patients. Although there was heterogeneity among the antibiotics administered, intraoperative redosing of antibiotic prophylaxis reduced the incidence of SSIs compared with a single preoperative dose of antibiotic prophylaxis regardless of the type of surgery. With regard to excessive blood loss, the ASHP/IDSA/SIS/SHEA joint guideline recommends redosing antibiotics in case of blood loss that exceeds 1.5 L [33].

### 3.6. Should SAP Be Prolonged after the Surgical Intervention?

SAP is aimed exclusively at preventing SSIs due to intraoperative wound inoculation and not infections that originate thereafter or in other locations. SAP should be administered and maintained at sufficiently high concentrations in serum, tissue, and the surgical site during the time that the incision is open but only while the incision is open. Erroneously, some surgeons believe that prolongation of SAP after the surgical procedure can protect the patient from postoperative infection.

Both the WHO global guidelines [7] and the CDC guidelines [8] recommend not prolonging the administration of SAP after surgical intervention to prevent SSIs. In 2020, a meta-analysis evaluating the effect of the postoperative continuation of SAP by de Jonge et al. [104] evaluated 83 relevant RCTs, of which 52 (19,273 subjects) were included in the primary meta-analysis. No conclusive evidence was identified for a benefit of postoperative continuation of SAP versus discontinuation. When best infection prevention practices were followed, the postoperative continuation of SAP did not add any additional benefit in reducing SSIs.

In a multicenter national retrospective cohort study published in 2019 [23], increasing the duration of antibiotic prophylaxis was associated with a higher risk of acute kidney injury and CDI, but did not lead to a reduction in SSIs.

In prescribing antibiotics for any indication, surgeons must always be responsible to handle antibiotics with care [105]. A list of principles has been identified that every surgeon must always respect in administering SAP (Table 3). 

## 4. Conclusions

Regarding SAP, surgeons should know to administer the “right” antibiotic for the “right” patient, at the “right” time, at the “right” dosage, and for the “right” duration. However, high rates of inappropriate SAP are common across the surgical pathway.

Many surgeons worldwide consider SAP of secondary importance in their clinical practice. The authors are aware that the problem related to good antibiotic prescribing practices is a global burden and that multidisciplinarity is the mainstay of the antimicrobial stewardship activities at the hospital level worldwide.

Adherence to SAP practices should be improved via multidisciplinary activities, both intraspecialty, actively involving surgeons, and interspecialty, directly connecting surgeons with other healthcare professionals, such as anesthesiologists, infectious diseases specialists, epidemiologists, microbiologists, hospital pharmacists, and pharmacologists.

According to a “peer-to-peer” approach, the article written by surgeons aims to address surgeons from all over the world hoping to raise awareness of SAP practices. The article is a narrative review shared by an international panel of experts aiming to contribute to the improvement of SAP in surgical units around the world but does not intend to replace the national and international guidelines cited in the text.

## Figures and Tables

**Table 1:** Characteristics of appropriate SAP [32].

	Characteristics
**Appropriate SAP**	Preventing surgical site infections. Improving patients’ outcomes. Reducing adverse effects: ○ Limiting opportunistic infections such as CDI. ○ Reducing risk of AKI. Reducing the consequences for patients’ microbiota. Limiting the emergence of antibiotic-resistant bacteria. Reducing the duration and cost of health care.

SAP: surgical antibiotic prophylaxis; CDI: *Clostridioides difficile* infection; AKI: acute kidney injury.

**Table 2:** Basic properties of the selected antibiotic for SAP [32].

	Properties
**Selected antibiotic**	Being active against the bacteria likely to contaminate the surgical site. Having the narrowest possible spectrum of activity. Being administered appropriately: ○ in an appropriate dosage and at the correct time. ○ for the shortest period possible. Being safe.

**Table 3:** Principles to be respected in administering SAP.

Questions	Principles
For which patients should SAP be administered?	SAP alone is unable to prevent SSIs and is not a panacea. There is no substitute for attention to infection prevention and control tactics, including preoperative preparation of the patients and meticulous hand hygiene practices. SAP is indicated for surgical procedures: ○ With a high rate of SSIs, or; ○ With prosthetic implants, or; ○ In patients with medical conditions associated with a higher risk of SSIs (i.e., immunosuppression or morbid obesity).
Which antibiotics should be chosen for SAP?	SAP should be effective against the aerobic and facultative/anaerobic pathogens most likely to contaminate the surgical site, including gram-positive skin commensals or normal flora colonizing incised mucosa. SAP in patients colonized or recently infected with MDR bacteria should be defined on a case-by-case basis. ○ When indicated for such patients, SAP should be based on the known susceptibility of the organism(s) and the patterns of antimicrobial resistance identified by the local hospital infection control committee. oABP, preferably in combination with mBP, is indicated in the prevention of postoperative complications after elective colorectal surgery. However, heterogeneity of antibiotic choice and treatment limits recommendation of specific agents.
When should SAP be administered?	SAP should be administered within 120 min, but ideally within 60 min, before incision to ensure adequate serum and tissue concentrations during the period of potential contamination. ○ Cefazolin may be administered via IV bolus 3–5 min before incision. ○ Vancomycin and fluoroquinolone, the use of which should both be rare, must be infused over a minimum of one hour.
How should the dose be chosen for SAP?	A single dose of SAP generally is sufficient. Especially, with respect to obesity, the dose depends on whether the agent is hydrophilic or lipophilic.
When should SAP be redosed intraoperatively?	Additional antibiotic doses should be administered intraoperatively for procedures that extend >2–4 h past the time of prophylactic administration (i.e., where duration exceeds two half-lives of the chosen antibiotic) or with associated major blood loss (>1.5 L).
Should SAP be prolonged after the surgical intervention?	There is no evidence to support continuing SAP postoperatively. The practice must be abandoned.

SAP: surgical antibiotic prophylaxis; SSIs: surgical site infections; MDR: multidrug resistant; oAPB: oral antibiotic bowel preparation; mBP: mechanical bowel preparation; IV: intravenous.

## Data Availability

Data sharing not applicable.

## Abbreviations

AMR	Antimicrobial resistance
CDI	Clostridioides difficile infection
CI	Confidence interval
HAIs	Healthcare-associated infections
IV	Intravenous
mBP	Mechanical bowel preparation
MDR	Multi-drug-resistant
MIC	Minimum inhibitory concentration
nBP	No bowel preparation
oABP	Oral antibiotic bowel preparation
OR	Odds ratio
RCT	Randomized controlled trial
RR	Risk ratio
SAP	Surgical antibiotic prophylaxis
SSI	Surgical site infection
Vd	Volume of distribution
